# Attenuation Impact on Acoustic Emission Signal Parameters in Damage Mechanisms Characterization of Composite Rebars

**DOI:** 10.3390/polym17233128

**Published:** 2025-11-25

**Authors:** Paweł Zielonka, Michał Smolnicki, Szymon Duda, Grzegorz Lesiuk

**Affiliations:** Faculty of Mechanical Engineering, Wroclaw University of Science and Technology, 50-370 Wroclaw, Poland; michal.smolnicki@pwr.edu.pl (M.S.); szymon.duda@pwr.edu.pl (S.D.); grzegorz.lesiuk@pwr.edu.pl (G.L.)

**Keywords:** acoustic emission, composite rebar, damage analysis, fracture behavior of composite materials

## Abstract

Composite materials have been extensively used across numerous industries due to their exceptional specific strength and corrosive resistance. However, ensuring their mechanical performance and structural integrity remains a critical challenge. This study provides an in-depth investigation into the damage mechanisms occurring in composite rebars manufactured via a modified pultrusion process, with a special emphasis on carbon, glass, and hybrid continuous fiber-reinforced polymers with epoxy resin matrix subjected to static tensile loading. To reveal the damage development, the acoustic emission (AE) technique was employed. Given the inherent complexity of composite microstructures, multiple failure modes can occur simultaneously, often masked by background noise and attenuation effects. Therefore, the core objective of this research is to evaluate and quantify the influence of acoustic attenuation on damage assessment in composite materials. This study introduces an optimization approach to minimize discrepancies between signals captured by different sensors, thereby enhancing the reliability of AE data interpretation. Results reveal that attenuation is strongly dependent on signal travel distance, frequency spectrum, and sensor type. Importantly, a data correction methodology is proposed to mitigate these effects, improving the accuracy of damage detection. Among the analyzed AE parameters, the initial frequency emerged as the most reliable feature for identifying the origin of acoustic events within hybrid composite structures. This finding represents a significant step toward more precise, attenuation-compensated acoustic emission monitoring, offering improved insight into failure mechanisms and contributing to the development of smarter diagnostic tools for composite materials.

## 1. Introduction

The market for composite structures has expanded during recent years. Thus, the structural integrity of the Fiber Reinforced Polymer (FRP) has been intensively investigated by researchers. This group of materials is challenging due to damage nature, i.e., various damage mechanisms such as fiber breakage, matrix cracking or delamination appear simultaneously and cause the fracture. The damage development depends on the material microstructure (i.e., layup configuration, constituents’ stiffness) and loading conditions [1,2,3,4]. Moreover, damage to composite materials begins to accumulate as soon as service operation starts (i.e., single fiber breakage). One of the methods for investigating damage development is stiffness reduction during structural lifetime, which can be easily determined on the specimen level. However, this method is phenomenological. It does not give any information about the actual damage mechanism [5,6,7]. On the other hand, damage accumulation in the real-size components is more challenging to determine, or in some cases, impossible. Growth rate of the number of acoustic emission events caused by various types of failures depends on the maintenance stage of the component and can be used as an indicator warning against sudden failure [8]. Due to the abovementioned reasons, it is crucial to investigate the material response in terms of the actual damage state, which, properly characterized can give information about the component degradation state.

Currently, many monitoring techniques are implemented to ensure safety and proper maintenance of structures such as bridges, pressure vessels or critical infrastructure [9]. The issue with phenomenological approaches and evaluation can be overcome by the implementation of non-destructive testing (NDT) on real-size components [10,11]. Thus, evaluation of structural integrity is increasingly verified using NDT methods to extend service of the composite components (in-service time can be increased with respect to the designing assumptions) and ensure their safety. Acoustic emission (AE) is one of the techniques which enables us to collect acoustic waves generated by damage events in material/structures using piezoelectric sensors bonded to the composite surface. Contemporarily, the examinations of the piezoelectric sensors embedded into structures were verified [12,13]. The outcome of the tests presents higher sensitivity of the mounted transducers and numerous advantages such as better sensor protection or eliminating the fixing effect than AE sensors attached to the surface. Currently, most of the AE applications in the in-service monitoring rely on continuous structural measurement and assessing the cumulative number of events collected during service. This procedure is widely used in the composite vessels monitoring to prevent medium leakage or structural failure [8]. Application of the deep learning methodology for real-time AE signal identification were developed to efficiently classify signals and distinguish them from the background noises [14]. Recently, the application of acoustic emission to determine specific damage mechanisms has been developed. Microstructural tests such as Single Fiber Fragmentation Test or mechanical experiments with dominated damage mode were applied to determine characteristic acoustic waveforms [15,16,17,18,19]. Due to the enormous amount of data during long-term measurements, machine learning techniques are used to cluster registered events [20,21,22,23].

In recent years, the use of composite rebars, such as glass fiber reinforced polymer (GFRP) and carbon fiber reinforced polymer (CFRP), has gained prominence due to their corrosion resistance, high strength-to-weight ratio, and long-term durability. Thus, the application of those members is highly required in civil structures, especially off shore, but not limited to those. It should be highlighted that composite rebars have been used in concrete deck or ground reinforcement (i.e., bridges) as well as anchoring. The performance of the composite rebars is presented in Refs. [24,25,26,27]. From the application point of view, and literature review, there are several aspects which affect the mechanical performance of those rebars, i.e., rebars–concrete adhesion, mechanical interlocking (in this case, overwrapping, which generates additional mechanical friction) [28].

Due to the inherent anisotropy, heterogeneity, and layered microstructure of composite materials, they present unique challenges for acoustic wave propagation and interpretation. A critical factor influencing the accuracy and reliability of acoustic measurements in composite rebars is the attenuation behavior of acoustic waves. Attenuation refers to the reduction in amplitude and energy of the wave as it travels through the material, which can result from mechanisms such as dispersion, absorption, and internal friction. In composites, attenuation is often frequency-dependent and strongly influenced by fiber orientation, resin properties, interfacial bonding, and manufacturing-induced defects [29]. Understanding and quantifying this attenuation behavior is essential for several reasons. First, it directly impacts the detectability of acoustic signals, especially those generated by damage mechanisms such as fiber breakage, matrix cracking, or delamination. Second, attenuation affects the signal interpretation, potentially hiding critical features or altering the apparent source characteristics. Third, reliable attenuation models are vital for localizing AE sources and for correcting signal amplitudes, ensuring that the measurements reflect material behavior rather than sensor or propagation artifacts. Furthermore, in practical applications, the coupling between the acoustic sensor and the composite rebar, as well as the sensor’s own frequency response, can further modulate the observed signal attenuation. Disentangling the contributions from the material and the sensor is therefore a prerequisite for accurate diagnostics [30].

In this context, a detailed investigation of the attenuation characteristics of acoustic waves in composite rebars not only enhances the fidelity of non-destructive evaluation techniques but also contributes to the development of more robust monitoring systems for advanced composite-reinforced structures. The relevant work objective is a determining impact of the damping effect on received AE events and the influence of implemented attenuation models on the composite rebars outcome. Performing tests on the hybrid composite structure with mechanical tests of basic components can deliver important data to compare the outcome variation in specific AE parameters on various reinforcement type materials. The outcome will be used to evaluate data improvement for further analysis of damage mechanism subjected to unidirectional fiber composites. Based on the current state of knowledge, establishing effect of attenuation on received AE events in hybrid composite rods were not performed. Obtained conclusions will be discussed in the following work.

## 2. Materials and Methods

### 2.1. Materials

Composite rebars investigated in this research have been manufactured by a modified pultrusion process (described in Ref. [31]), reinforced by unidirectional continuous fiber reinforcement with polymeric matrix. In the following work, three components, anhydride cured, thermosetting epoxy matrix from Sika company in Baar, Switzerland were used in the manufacturing process. The polymer formulation and mixing ratio are presented in Table 1.

Reinforcement used in the composite rebars is based on glass fiber, carbon fiber and their mixing as a hybrid interlayer structure. The datasheet properties provided by the manufacturer are shown in Table 2.

**Figure 1:**
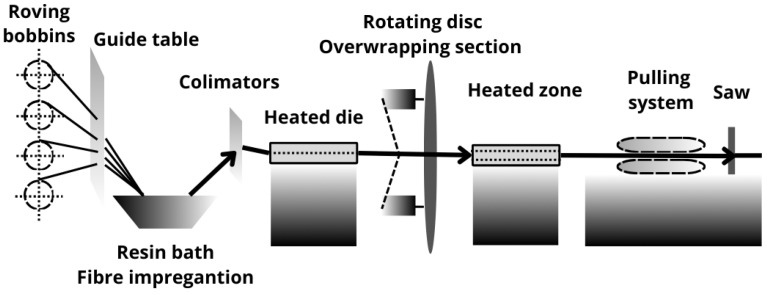
Pultrusion process used for composite rebars manufacturing.

**Figure 2:**
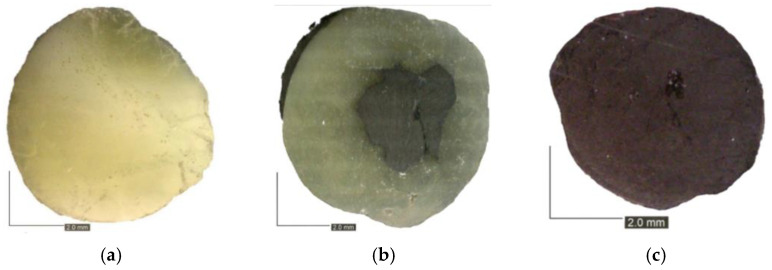
Cross-section of composite rebars conducted under experimental campaign (**a**) Glass fiber rebar with nominal diameter 8 mm; (**b**) Hybrid fiber rebar with nominal diameter 8 mm; (**c**) Carbon fiber rebar with nominal diameter 6 mm.

The modified pultrusion process presented in Figure 1 was used to manufacture composite rebars for concrete reinforcement. The process is based on pulling impregnated reinforcement through the heated die, which consolidates the fibers and reduces porosity and resin volume content. The gel stage was obtained at the end of the heated die. The unidirectional reinforcement was overwrapped by radial 800 Tex carbon fiber tows to squeeze the rod and acquire a developed cross-section. Modification provides higher adhesion of the component to the concrete compared to the traditional pultrusion process, which allows for achieving only smooth-symmetrical profiles. The cross-section of the manufactured profiles is presented in Figure 2. Each of the rebar types is characterized by a high fiber volume ratio (around 65%) and a porosity level up to 5% established using µCT scan.

The main parameters responsible for a proper curing process were temperature at heated zones and linear velocity of pultruded rod. The length of the heated die was 1 m, while the heated zone, which is characterized by non-contact heat transmission, was 3 m. The temperature setup is characterized by higher inertia of the system, which determines setting heated section variables at first. After system stabilization, the velocity adjustment was applied to achieve correct rebars properties. The final parameters used in the process are shown in Table 3.

### 2.2. Acquisition System and Parameters

Acoustic wave measurements were performed using a Vallen AMSY-6 acquisition system and a pair of piezoelectric sensors. The acquisition parameters setup applied in the system is shown in Table 4.

Measured acoustic emission signals are composed of a spectrum of frequency. Extraction of frequency (e.g., the frequency center of gravity or peak) of the specific acoustic events can be achieved using Fast Fourier Transform (FFT). The parameters used in the extraction process are presented in Table 5. Another method is to divide the number of signal cycles by the period of time. The parameters such as initiation frequency FI or average frequency AF were established in this manner.

**Figure 3:**
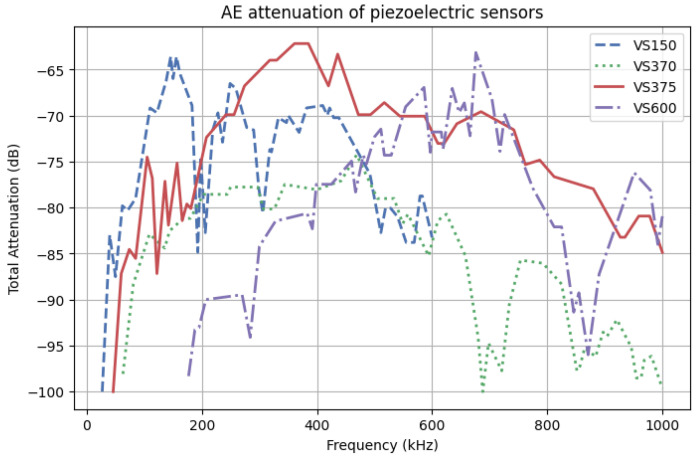
Attenuation characteristics of piezoelectric sensors based on the certificates provided by the manufacturer.

A crucial aspect during acoustic events registration is the selection of sensors. The recorded response by the sensor is highly dependent on the frequency of the event. The sensors can be designed to be the most sensitive at a specific resonant frequency or to provide a flat frequency response over a wide frequency range. The characteristics of the applied sensors are in Figure 3 and Table 6.

Based on Figure 3, the characteristics can be analyzed, providing information about the mentioned peak sensitivity, which in this case is 375 kHz. The attenuation profile is characteristic for each individual sensor and determined after the manufacturing process as a calibration table. The type of the sensing element needs to be chosen for the expected range of frequencies characteristic of analyzed objects or crucial information will be damped.

### 2.3. Experimental Method

**Figure 4:**
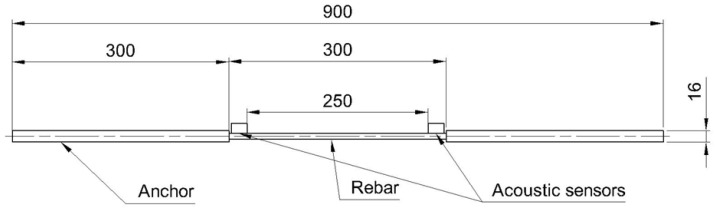
Composite rebar specimen with mounted AE sensors prepared for static tensile loading.

The experimental campaign aims to verify the impact of the acoustic wave attenuation on the registered data and the outcome resulting from the proposed method. Analysis of acoustic signals was performed for data obtained from static tensile tests of composite rebars with various types of reinforcement following the ASTM D7205 standard [32]. To prepare composite rebars for static tensile tests, the specimens were anchored in the steel tubes using an epoxy injection anchor. Two AE sensors were glued on the opposite side of the measurement length to apply the localization processor during the tests. The dimensions of the setup are presented in Figure 4. To characterize the AE events specific to each set of rebars, three specimens for each of three reinforcement types were analyzed.

**Figure 5:**
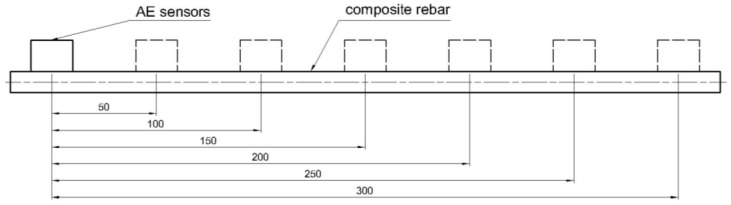
Attenuation characterization of various types of fibrous reinforcement carried out with the pulsing procedure. The localization of sensors was highlighted in the figure—the first sensor with solid lines and various positions of the second sensors with dashed lines.

Moreover, the influence of the measurement length on the registered value of various AE parameters was analyzed according to ASTM E976 standard [33]. As a signal source, pairs of the three sensor types (VS150, VS370, VS600) were glued at different distances, and the pulsing procedure to generate repeatable acoustic wave was performed. The sensors have been reassembled at each transducer distance, to take into account the mounting effect on the results as given in Figure 5 and obtain 10 values for error estimation. The sensors were fastened with repeatable force and lithium grease as a transmission medium based on recommendations in ASTM E650 [34].

An acoustic emission system collects a significant number of events during the mechanical testing of composite materials. The aim of the conducted analysis is to determine the characteristic set of AE parameters, which can describe damage mechanisms under loading conditions. To overcome the challenge, the following assumptions were used causing limitation of the AE data shown on Table 7:

1. Locations between sensors—events registered during tests occur not only from the damaged material, but also from phenomena such as friction of the gripping system, damage in the anchor, hydraulic unit noise (power unit for testing system). This rule provides confidence as to the origin of the events. The data collected between the sensors’ positions were taken to analysis.
2. Hits equal to one—sometimes the acoustic waves can overlap, and the AE system registers several hits as one event. Eliminating the data with more than one hit was introduced to ensure proper investigation of parameters characterized by a specific occurrence.

To characterize a specific AE event, the following features were utilized:

- Amplitude A—peak amplitude of the hit, maximum value measured in mV. It is often expressed in dB scale using the following formula:

(1) $$A\;\left[dB\right]=20*{log}_{10}(A\left[mV\right])$$

- Energy E—integral of the squared AE-signal over time. It represents the total energy content of the acoustic wave that was detected.

(2) $$E\;\left[dB\right]=10*{log}_{10}(E\left[eu\right])$$

- Initiation frequency FI—calculated by dividing Counts to Peak (CTP) by Rise Time (RT). CTP is the number of positive threshold crossings from the start of the hit to peak amplitude. RT is calculated as the time between the start of the hit and the peak amplitude.

(3) $$FI=\frac{CTP}{RT}$$

- Reverberation frequency FR—calculated by dividing the difference in CNTS (cumulative number of positive threshold crossings of a hit) and CTP by the duration of the hit, less of rise time. The parameter is calculated by the following equation.

(4) $$FR=\frac{CNTS-CTP}{Dur-RT}$$

- Rise Angle RA—defined as the ratio of rise time and peak amplitude. Amplitude was reduced by a threshold value specific to each measurement and the background noise characteristic for the measurement environment [35].

(5) $$RA=\frac{RT}{A-Thr}$$

- Average Frequency AF—determined as the ratio between the ring counts and duration. Correlation of the RA-AF is proposed by Japan Concrete Institute (JCI) as a standardized method JCMS-IIIB5706 [36] for tensile and shear crack classification in concrete structures [35].

(6) $$AF=\frac{CNTS}{Dur}$$

- Falling Angle FA—calculated as the ratio of duration–rise time difference and difference between amplitude and rise time.

(7) $$FA=\frac{Dur-RT}{A-Thr}$$

**Figure 6:**
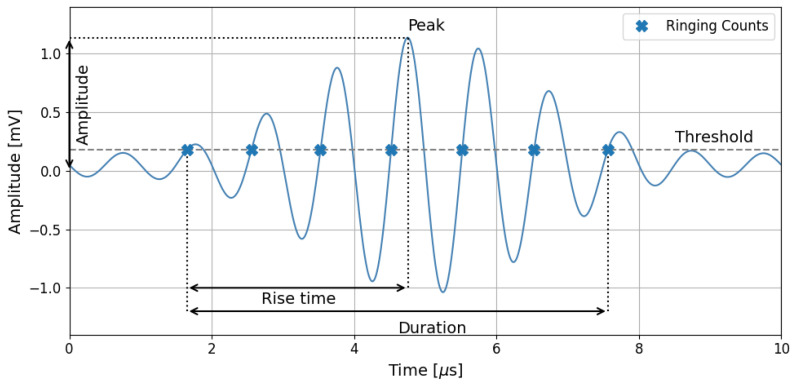
Exemplary acoustic waveform with characteristic parameters.

An exemplary acoustic wave with highlighted basic parameters is presented in Figure 6. The threshold level is marked on the figure, which represents the level of the measured signal, recognized as the beginning of the acoustic wave. The threshold value is selected based on the environmental background noise, typically 3 dB over registered signals.

**Figure 7:**
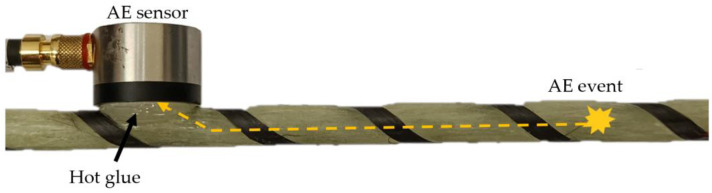
Acoustic wave path from the damage location to the piezoelectric sensor. In the investigated case, the AE signal has to travel through different materials such as matrix, reinforcement, hot glue and sensor material.

Acoustic waves registered by the piezoelectric sensors differ from the original acoustic signal. It is highly dependent on the signal transfer medium, material imperfections such as porosity, internal cracks or components used for acoustic transmission (hot glue, grease). The shape of the analyzed component (rod or sphere) and the internal microstructure are crucial to investigating acoustic wave dissipation and attenuation. Taking into account the following phenomena, it would explain the situation where the same damage event registered by the AE system by various sensors is characterized by different parameters. The path of the acoustic wave is highlighted in Figure 7.

### 2.4. Amplitude and Energy

To overcome the aforementioned issues, the attenuation analytical Equations (8) and (9) with optimization procedure were proposed to normalize received parameters. The method is based on randomly selected values from the range of parameters used in the attenuation equations. The proposed method was chosen due to multiple applied variables with the uncertainty of the function continuity and the occurrence of local minima. This approach allows us to obtain a more reliable final output. The optimization convergence was checked by verification of the best-found value changes over recent iterations following Equation (10). The window size k for looking for better value during optimization was set to 5000 iterations and variations tolerance ϵ to 0.0001. Maximum numbers of conducted iterations were limited to 100,000 iterations.

(8) $$A_{0}=A/[e^{-\alpha_{A}*f^{p}*x}*x^{-n_{A}}*e^{-\alpha_{Am}*f*t}*t^{-n_{m}}*T*Z]$$

(9) $$E_{0}=E/[e^{-{2\alpha }_{E}*f^{p}*x}*x^{-n_{E}}*e^{-{2\alpha }_{Em}*f*t}*t^{-n_{m}}*T*Z]$$

- $A_{0},\;E_{0}$—initial amplitude and energy of the acoustic wave
- A, E—registered amplitude and energy using a piezoelectric sensor
- $\alpha_{A}{,\;\alpha }_{E}{,\;\alpha }_{Am}{,\;\alpha }_{Em}$—coefficient of amplitude and energy attenuation over distance, determined for an investigated material and mounting system (hot glue, grease)
- *f*—frequency center of the gravity
- *p*—parameter specific to the material, characterizing nonlinear correlation of attenuation with increased frequency, equal or higher than one
- $x^{-n_{E}}$—position of the damage event based on the localization processor raised to the $n_{A}$ or $n_{E}$ power dependent on component shape and microstructure, describe dependence of the registered value with AE source distance
- $t^{-n_{m}}$—thickness of the mounting system of the piezoelectric sensor raised to the $n_{Am}$ or $n_{Em}$ power dependent on mounting shape
- *T*—transmission coefficients between composite rebars/mounting system/sensor
- *Z*—attenuation of the piezoelectric sensors depended on the frequency spectrum of the acoustic wave. Z value is calculated based on the frequency center the gravity of the registered AE signal, and the attenuation profile of AE sensors (Figure 3)

(10) $$|{score}_{best}\left[i\right]-{score}_{best}\left[i+k\right]|<\epsilon$$

**Figure 8:**
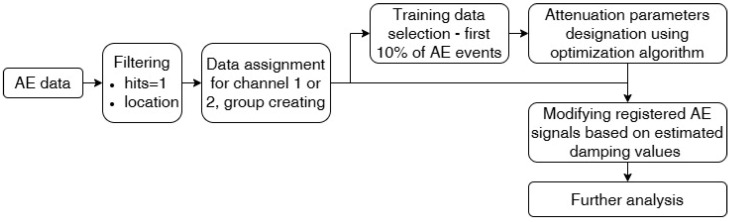
Algorithm of the attenuation implementation in acoustic emission events.

Reduced data, which fulfills location and hits limitations, are taken into investigation. Loading conditions in the material can change the attenuation properties due to crack development or fiber breakage, so the first 10% of reduced data were taken for the optimization procedure. Training data were grouped into sets of data based on whether they were registered by channel 1 or channel 2. To characterize the attenuation output of applied parameters for each iteration, the score was calculated using Equations (11) and (12). Designated attenuation parameters were used to modify the whole AE data for further analysis. The algorithm is presented in Figure 8. The reproducibility of the algorithm outcome was verified by running the optimization procedure 10 times. If the coefficient of variation were under 2% for the achieved results, the mean value was qualified as the correct solution.

### 2.5. The Attenuation Coefficient

In the perfect material with ideal sensor coupling, without dispersion and damping effect, the signal registered by both channels will be the same. To describe the mismatch of attenuated results and compare them, dividing the amplitude or energy values collected by channel 1 to data from channel 2 was proposed by Natalie Godin [37]. To take into account the nonlinear behavior of the attenuation characteristic, the obtained proportions were logarithmic. Additionally, if the proportion were reversed, the logarithm would have a negative value with the same modulus. To standardize both cases, when the numerator is higher or lower than the denominator, the modulus of proportion was taken. The division was considered a better option due to the lower impact of the initial level of amplitude or energy on the outcome than subtraction. For higher values, the subtraction outcome will have a higher influence on the summary of the training data score. The main aim was to minimize the obtained score during simulation and save the best attenuation parameters.

(11) $$score=\sum_{i=1}^{n}\left|{log}_{10}(A_{01}(n)/A_{02}(n))\right|$$

(12) $$score=\sum_{i=1}^{n}\left|{log}_{10}(E_{01}(n)/E_{02}(n))\right|$$

To achieve a reliable outcome, the estimated amplitude or energy cannot be lower than the received signal values.

### 2.6. Frequency and Waveform Slope Parameters

To exclude attenuation impact on the frequency and waveform slope parameters, the mean value of the variables registered by two sensors was proposed. The concept of the applied formula was that AE event parameters registered near the first sensor will be compensated for by the extensive distance to the second sensor. In the middle section, recorded data should be characterized by the same variables, which can be different due to various mounting conditions. The obtained outcome can be compared between specimens distinguished by the same acquisition setup and specimen. The following attenuation equations were proposed:

(13) $${FI}_{0}=({FI}_{1}+{FI}_{2})/2$$

(14) $${FR}_{0}=({FR}_{1}+{FR}_{2})/2$$

(15) $${RA}_{0}=({RA}_{1}+{RA}_{2})/2$$

(16) $${AF}_{0}=({AF}_{1}+{AF}_{2})/2$$

(17) $${FA}_{0}=({FA}_{1}+{FA}_{2})/2$$

The authors take into account that designated values cannot be treated as the real characteristic of the generated initial waveform, which needs further research. The following elaboration supports the analysis of the AE results, reducing the influence of the applied mounting conditions and the location of the events.

## 3. Results and Discussion

In the following section, the outcome of the assumption made is presented. The results will be divided into three subsections: amplitude and energy, frequency, and waveform slope, to discuss the modification of the variables after the attenuation process.

### 3.1. Amplitude and Energy

**Figure 9:**
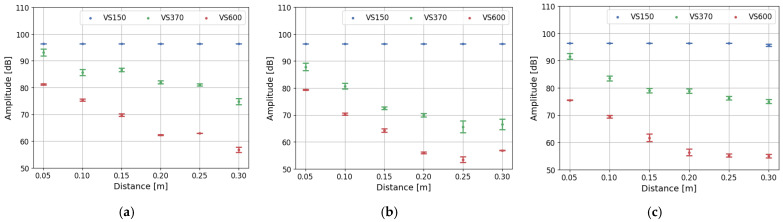
Dependency of the registered amplitude on the distance between piezoelectric sensors during pulsing procedure for (**a**) glass fiber rebars, (**b**) hybrid rebars, (**c**) carbon fiber rebars.

**Figure 10:**
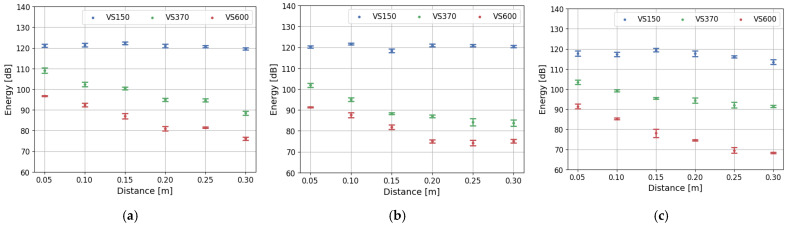
Dependency of the registered energy on the distance between piezoelectric sensors during pulsing procedure for (**a**) glass fiber rebars, (**b**) hybrid rebars, (**c**) carbon fiber rebars.

The amplitude and energy of acoustic emission signals are strongly influenced by distance and frequency due to the nature of how AE waves propagate through a material [38]. In view of the geometrical spreading and material damping, the strength of AE waves is absorbed and decreases with rising distance. The shape of the component, which is correlated with the AE wave shape, influences the energy distribution over a larger area. The spectrum of the frequency characteristic for the discussed AE wave is also crucial due to stronger attenuation of the higher frequency range than the low-frequency ones caused by scattering from microstructures (e.g., grains, inclusions) and viscoelastic damping. Lower-frequency AE signals retain amplitude better over long distances. Also, piezoelectric sensors are crucial for signal registrations due to their ability to convert mechanical stress or deformation into an electrical voltage. The impact of specific sensors frequency-dependent sensitivity on registered data is discussed by Godin’s work, which highlights divergence in the tensile tests AE outcome collected by nano30 and pico HF transducers mounted on the same position on specimen [30,39]. Correct selection of the transducers provides for registering data of particular significance. The importance of the sensor selection is corelated with the damping characteristics compared in Figure 3, which highlights attenuation dependency with the event frequency spectrum various for different piezoelectric sensors. Figure 9 and Figure 10 present the value of amplitude and energy measured by three types of sensors in the investigated composite structures during the pulsing method. As is apparent, the signal registered by the VS150 sensor presents no visible signs of amplitude and energy weakening in the discussed distance range. Signals recorded by VS370 and VS600 piezoelectric sensors are highly dependent on the detector span. The high-frequency signal has the highest attenuation among the discussed sensors.

Similar test outcomes are achieved by Jung in the study of woven composite specimen, recording AE events by the sensors mounted in the various distance from the acoustic source [40]. The number of registered signals decreases with increasing transducer distance. Most of the variations can be noticed in measured high frequency events. The authors suggest applying sensors in the range of 20–40 mm to have reliable data, which is impractical for structural monitoring of composite components. Such distance has no effect on the frequency characterized AE data. The material of the composite rebar has no significant effect on signal attenuation. A slightly higher attenuation can be noticed for the VS370 sensor in the hybrid rebar compared to the values of amplitude and energy registered in glass and carbon fiber structures. Sensors’ localization and selection need to be conducted based on the microstructure knowledge, expected AE events parameters and targeted measurements. Hafizi examined the attenuation impact on the unidirectional glass fiber reinforced polymer under various angles, using breaking the pencil method as an acoustic emission source [41]. The importance of piezoelectric sensors was confirmed, presenting significant differenced in the registered signal by broadband and resonance frequency sensor. The impact of the distance on the frequency spectrum after Fast Fourier Transform was highlighted. The recommendation of the maximum radius of approximately 200 mm was performed. The mentioned manuscripts confirm the significant influence of the distance and piezoelectric sensors on the parameters received from the tests; however, most of the conducted research has been concentrated on laminated plates, not composite rods.

To overcome the complexity of the AE wave attenuation nature, the optimization method was utilized based on Equations (8) and (9) and the proposed score. The boundary conditions for the simulations are described in Table 8.

Boundary conditions presented above are either based on the literature [42,43,44] or were determined by taking into account physical aspects of phenomena. For example, transmission between the boundaries of materials caused attenuation of the signal; therefore, the accepted range of values was assumed to be (0, 1). In the case of geometrical spreading, the coefficient 1.5 is the theoretical limit for spherical structures with signal scattering, while 0 is reserved for ideal 1D geometry. The number of performed iterations and the convergence of the best result based on Equations (11) or (12) were tracked for each specimen.

**Figure 11:**
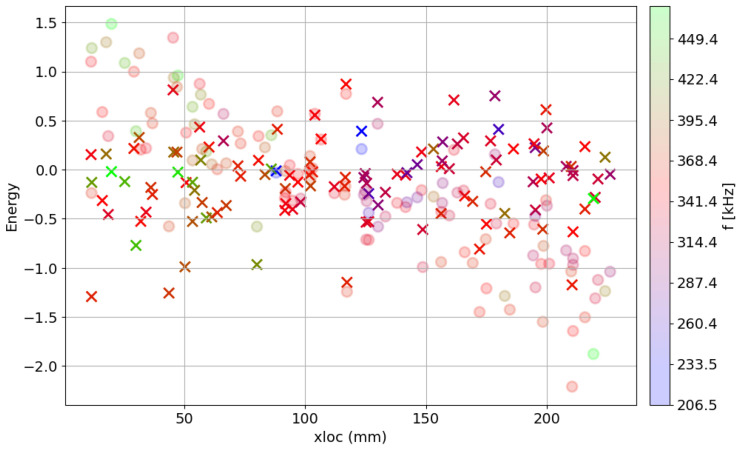
Correction of the training dataset over localization on the rebar. The frequency center of gravity for each event is highlighted. Transparent circles were used to mark the original data, while the crosses were applied for AE events after correction using obtained damping parameters.

**Figure 12:**
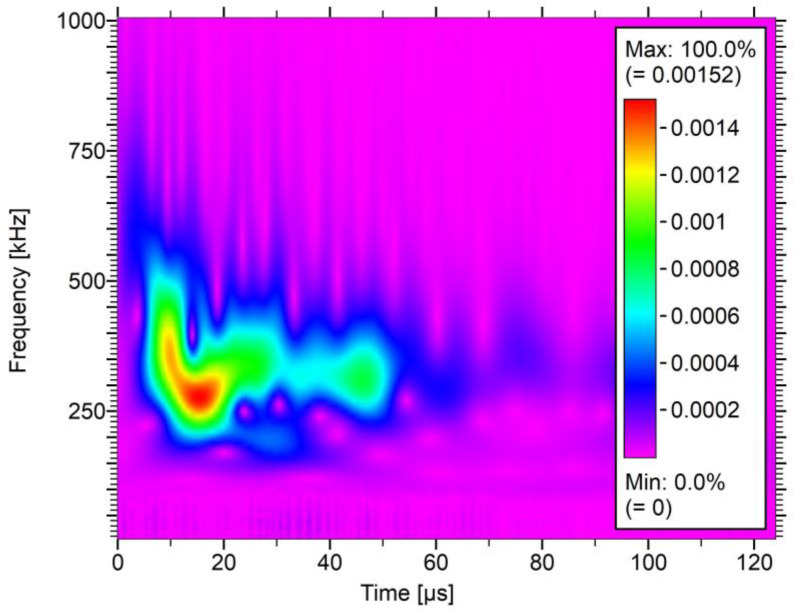
Frequency distribution of the AE signal over the time domain.

**Figure 13:**
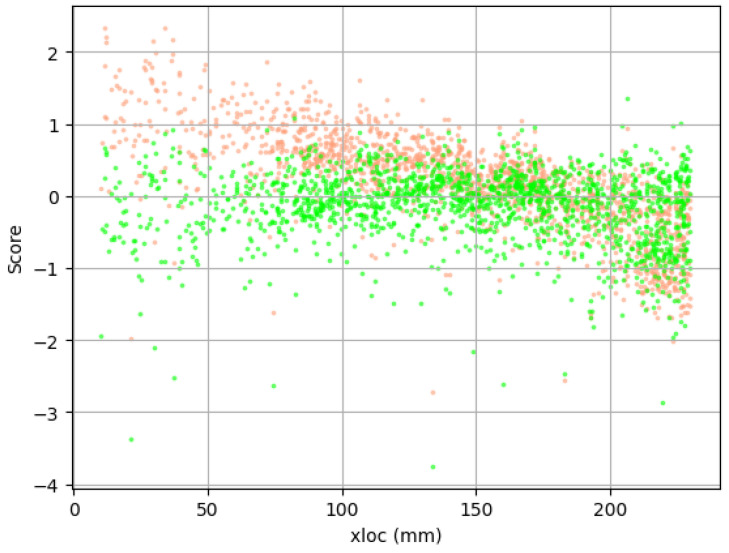
Correction of the whole dataset based on values designated using the training set. The ratio of the data obtained from both sensors on a logarithmic scale was presented over localization on the rebar. The green markers were used for attenuated results, the orange ones for the recorded data.

The training dataset was used in the simulation to achieve the best results of attenuation. The outcome from one of the simulations is provided in Figure 11 presenting the value of the individual score over the location of AE event. The attenuated values of amplitude and energy were plotted over the transparent original data to better compare the outcome. The obtained results are not characterized by perfect matching of the attenuated signals; some scatter of the data can be noticed. There can be multiple reasons for such phenomena, such as the acoustic emission signal consisting of a spectrum of various frequencies or anisotropy of attenuation parameters in composite material—different coefficients along and perpendicular to the fibers. The spectrum of frequency of one of the registered acoustic waves, after Fast Fourier Transform, in the time domain is shown in Figure 12. The coefficients established from training data were used in the correction of the whole set of AE signals. One of the corrected databases is presented in Figure 13. Attenuation impact is visible on the cited data. Used correction helps with eliminating the mounting influence or localization on the results.

The improvement of the score value used as an indicator of disagreement for AE data is presented in Table 9 and Table 10. The results are shown as a mean value of the cost function divided by the number of events taken into the training dataset. Based on that outcome, the glass fiber rebar has the best initial score and score after optimization process among all rebar types. This means the parameters extracted from channel 1 and channel 2 are the most similar to each other. The worst indicator was achieved for carbon fiber profiles. The distribution of the results can suggest that the carbon material had been more attenuated than glass fibers, which can be a reason for the higher differences between sensors. The hybrid rebars indicators were in the middle, closer to the carbon fiber outcome.

The attenuation parameters for each composite structure were designated and collected in Table 11. The most repeatable results were obtained for amplitude optimization in glass fiber rebars. The values achieved for energy parameters were divergent. For the following analysis of the structural damage, the amplitude outcomes are recommended to be used.

Based on the results obtained from the conducted analysis, the attenuation is one of the key factors having a crucial impact on the analysis amplitude and energy data. The proposed method needs further improvement to achieve outcome with less scatter of the data and more reliable results. The following modifications were proposed to enhance the algorithm:

- Reduce the range of the boundary conditions accepted to the simulation based on preliminary studies, such as estimation of the attenuation coefficient of hot glue or the transmission ratio between different mediums
- Improve the optimization algorithm by using, for instance, a gradient descent-based approach to obtain better convergence of the data
- Implement further analysis of the sensor attenuation impact on the results. The frequency center of the gravity parameter obtained from the FFT algorithm does not reflect the whole complexity of the phenomena as present in Figure 12 which can have a significant influence on the energy optimization process.

### 3.2. Frequency

**Figure 14:**
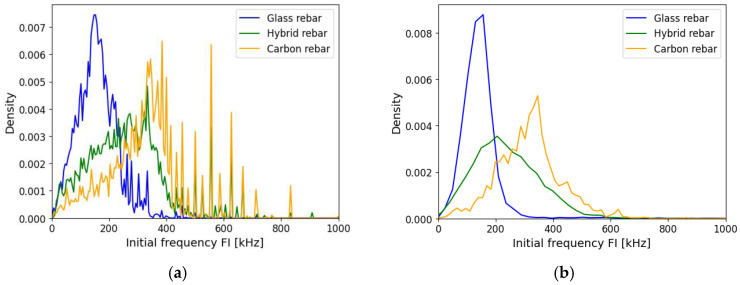
Initial frequency FI of composite rebars, (**a**) registered data, (**b**) AE data after applying attenuation assumption.

Frequency is one of the elementary parameters used for acoustic wave description. In general, the FFT analysis is conducted to obtain the variables, such as the frequency center of gravity or the maximum amplitude of the frequency spectrum. In the following work, the other frequency parameters obtained from the analysis of the raw acoustic signal registered by the AE system are described. The initial frequency FI is obtained as the number of counts registered in the period of time to the peak value. The parameter can be affected by the threshold value set as an acquisition setup at the beginning of the test, determining the moment of counts and time registration. The impact of the proposed attenuation method, described with Equation (13), is shown in Figure 14. The application of various reinforcement materials has a significant impact on the initial frequency distribution registered by the AE system. The values of frequency characterized by glass and carbon fibers present a slight overlap in the 200 kHz frequency, but most of the events were divided. The attenuation method improves the outcome from the measurements, reducing the number of events that present the same range of frequency values. The hybrid rebar represents mixing of the values characteristic for two types of reinforcement, which suggests that damage mechanisms occur in both reinforcement materials.

**Figure 15:**
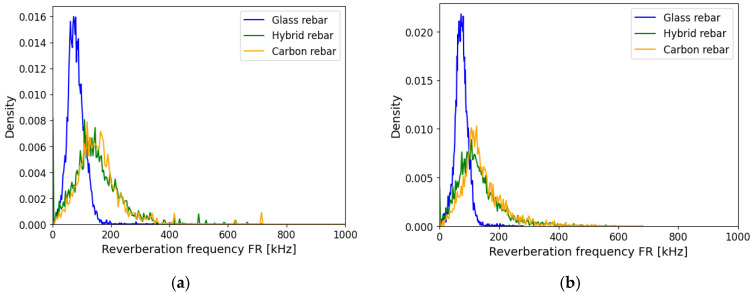
Reverberation frequency FR of composite rebars, (**a**) registered data, (**b**) AE data after applying attenuation assumption.

The reverberation frequency was determined after the amplitude peak of the signal. The attenuation method from Equation (14) was not modifying the obtained raw data as shown on Figure 15. The events were more concentrated in the center of gravity of the distribution. The shifting of the outcome from glass and carbon reinforcement can be noticed. The result of the hybrid and carbon rebar was excellently overlapped.

**Figure 16:**
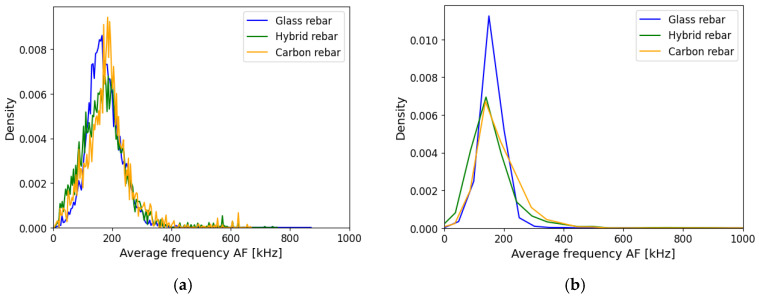
Average Frequency AF of composite rebars, (**a**) registered data, (**b**) AE data after applying attenuation assumption.

The average frequency AF is a highly used parameter in the concrete mechanism characterization to select the cracks subjected to tensile/shear mode or a combination of them—mixed mode [36]. The AF distribution of registered data was similar for each type of specimen. The attenuation assumption did not change the arrangement of the AE data as illustrated in Figure 16.

Concerning the unidirectional fiber reinforced polymer structure, the proposed attenuation method has the greatest impact on the initial frequency distribution values. The mentioned parameters also represent the highest distinction with respect to the results obtained, which can be applied for hybrid composite structure damage characterization. Other parameters were not highly affected by the proposed assumption.

### 3.3. Waveform Slope

**Figure 17:**
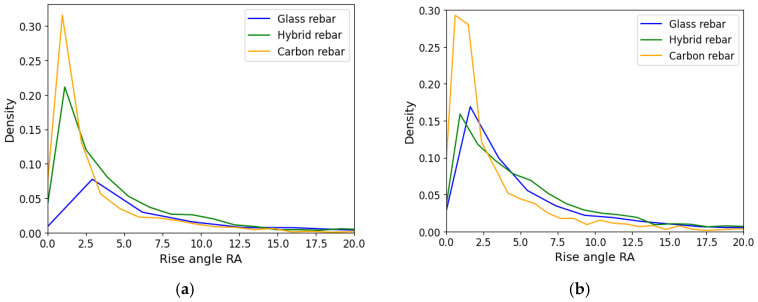
Rise angle RA of composite rebars, (**a**) registered data, (**b**) AE data after applying attenuation assumption.

**Figure 18:**
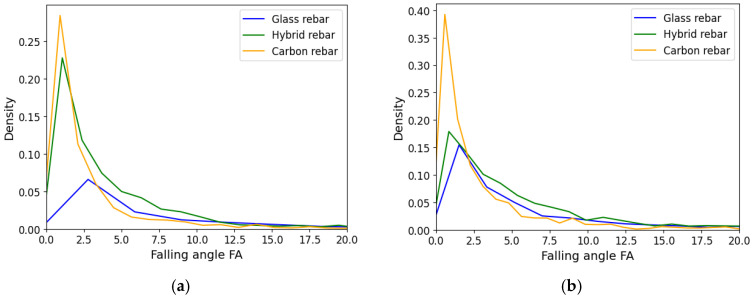
Falling Angle FA of composite rebars, (**a**) registered data, (**b**) AE data after applying attenuation assumption.

The RA and FA values defined the shape of the registered acoustic wave. The rise angle was determined by the ratio of the rise time and peak amplitude value, while the falling angle was the period from the peak amplitude to the end of the signal to the maximum amplitude value. The low range of the results are assigned for tensile breakage caused by the short duration of the event and higher frequency. The higher results values were delivered by shear loading modes due to longer rise time and lower frequencies [36]. The distribution of the rise angle and the falling angle is presented in Figure 17 and Figure 18. In both cases, the carbon fiber rebars are characterized by lower values of RA and FA. The distribution of hybrid data was slightly shifted to the carbon fiber center of gravity of the data, which can be affected by carbon fiber breakage. The data from hybrid and glass fiber profiles after the attenuation process are more similar than before in both cases. The dominance of the carbon fiber in the low values region has been preserved.

**Figure 19:**
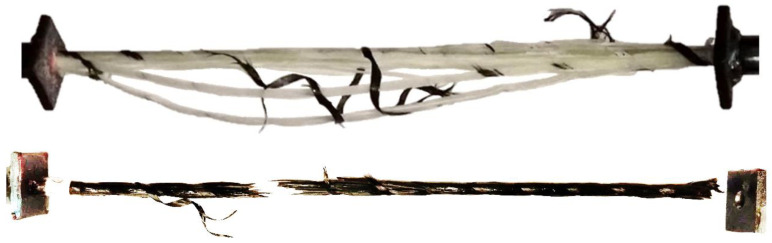
Glass fiber (**up**) and carbon fiber rebars (**bottom**) after experimental static tensile test.

The features distribution is highly compliant with damage behavior subjected to composite rebars. The carbon fiber composites were characterized by sudden failure, while glass fiber structures were subjected to continuous damage development, delamination between unidirectional tows and finally failure. In the interlayer hybrid composite, the delamination on the material boundary is common due to differences in the mechanical properties of various reinforcement types. The differences in the final rupture of the rebars is presented in Figure 19.

In the forward step, the obtained results will be applied to specific damage mechanism characterization of composite rebars under static and fatigue tensile loading conditions. The impact of attenuation will be analyzed in the case of cluster determining and structural condition assessment. Also, improvement of the proposed method is assumed when data quality improvement is observed during the clustering process.

## 4. Conclusions

The influence of the attenuation phenomena on the obtained AE parameters in the composite rebars reinforced by various types of fibrous material under static tensile loading conditions were considered within the research framework. The following conclusions were made on the basis of the conducted experiments and numerical optimization campaigns.

1. Localization of the acoustic emission source, waves spectrum of frequency or applied piezoelectric sensors have effect on registered amplitude and energy value of the same AE event, which makes difficult the correct interpretation of data intended for damage mechanism classification.
2. Numerical optimization process provides for reduction of the discrepancy of the AE results, assuming the range of the damping parameters characterized attenuation equations. The numerical approach ensures obtaining more reliable variables characterizing registered AE events. The score value characterizing amplitude and energy mismatch of the registered acoustic data by two piezoelectric sensors was reduced by 33% for glass fiber rebars and 40–50% for hybrid and carbon fiber structures.
3. The initial frequency is selected as the best parameter for characterization of the acoustic emission origin in the hybrid composite structure. Shifting of the variable distribution for the glass and carbon fiber reinforcement will be used for further damage mechanism classification under cyclic tensile loading conditions to characterize dominant damage mechanisms in the specific lifetime stage.
4. The attenuation process has an impact on the rise angle RA and falling angle FA parameters distributions for glass and hybrid composite structures, which have similar dispersal after processing.
5. The visual observation of the damage evolution in the composite rebars under tensile loading provides for defining dominant fracture behavior during testing. The glass fiber structure is characterized by developed crack evolution with delamination area through bundle interface; therefore, carbon fiber rebar is more brittle, with sudden failure. The damage types were highlighted in the specimen images.

## Figures and Tables

**Table 1:** Overview of matrix components used in the experimental campaign.

	Biresin CR141 Resin (A)	Biresin CH141 Hardener (B)	Biresin CA141 Accelerator (C)
Components	≥90% bisphenol-A-(epichloryhydrin) epoxy resin (number average molecular weight ≤ 700)	≥50%–≤100% tetrahydromethylphathalic anhydride	≥50%–≤100% 1-methylimidazole
Mixing ratio, parts by weight	100	90	2

**Table 2:** Applied reinforcement types during experimental campaign.

	Glass Fibers	Carbon Fibers
Manufacturer	StarRov	Zoltek
Type of sizing	silane	silane
Density [g/cm^3^]	2.60	1.78
Tensile strength [MPa]	1765	4137
Modulus of elasticity [GPa]	74	242
Strain at failure [%]	-	1.5
Fiber diam. [µm]	23	7.2
Tex	4800	3600

**Table 3:** Technological parameters controlled during modified pultrusion process.

Temperature [°C]						Velocity	
Bath	Die1	Die2	Zone1	Zone2	Zone3	Linear [m/min]	Radial [1/min]
60	110	125	160	150	140	0.3	1.25

**Table 4:** Acquisition parameter.

Parameter Name	Parameter Value
Threshold [dB]	35
Rearm time [µm]	400
Duration discr. time [µm]	200
Amplifier	AEP5 (34 dB)
Preamplifier gain [dB]	34
Pre-trigger samples	100 (10 µm)
Post-duration samples	100 (10 µm)
Frequency filter [kHz]	95–850

**Table 5:** FFT-feature extractor.

Parameter Name	Parameter Value
Start sample relative to trigger sample	−100
No. of samples for extraction window	256
No. of samples for FFT calculation	4096

**Table 6:** Overview of piezoelectric sensors used in the study.

	VS150-M	VS370-A2	VS375-M	VS600-Z1
Frequency range [kHz]	100–450	170–590	250–700	550–730
Frequency of sensitivity peak [kHz]	150	370	375	600
Capacity [pF]	350	47	390	200
Size(dxH) [mm]	20.3 × 14.3	8.5 × 13.0	20.3 × 14.3	4.75 × 5.8

**Table 7:** Impact of the applied limitations on the analyzed number of acoustic emission events per specimen.

	Number of AE Events per Specimen			Number of AE Events per Specimen After the Reduction Scheme		
	Mean	SD	CV	Mean	SD	CV
Glass fiber rebar	396,080	28,404	7.2	6852	474	6.9
Hybrid fiber rebar	71,991	1674	23.3	1707	364	21.3
Carbon fiber rebar	49,560	14,307	28.9	1086	380	35.0

**Table 8:** Boundary conditions used in optimization.

Parameter		Min Value	Max Value	Unit
Material attenuation	$\alpha_{A},\;\alpha_{E}$	0.001	1	Np/mm/kHz
Mounting material attenuation	$\alpha_{Am},\;\alpha_{Em}$	0.01	10	Np/mm/kHz
Geometrical spreading	$n_{A},\;n_{E}$	0	1.5	-
Geometrical spreading of mounting	$n_{m}$	0	1.5	-
Frequency power	pot	1	2	-
Glue thickness	t	0.1	5	mm
Transmission	T	0	1	-

**Table 9:** Improvement of the mean score value per event after optimization for amplitude value.

	Glass Rebar			Hybrid Rebar			Carbon Rebar		
	Mean	SD	CV	Mean	SD	CV	Mean	SD	CV
Initial score	0.22	0.01	3.5	0.28	0.06	21.9	0.30	0.04	13.0
Best score	0.15	0.02	14.9	0.17	0.02	13.3	0.18	0.03	18.6
Improvement	32%			39%			40%		

**Table 10:** Improvement of the mean score value per event after optimization for energy parameters.

	Glass Rebar			Hybrid Rebar			Carbon Rebar		
	Mean	SD	CV	Mean	SD	CV	Mean	SD	CV
Initial score	0.36	0.05	14.8	0.51	0.10	20.5	0.58	0.11	19.1
Best score	0.24	0.02	9.5	0.26	0.05	17.2	0.28	0.06	21.4
Improvement	33%			49%			52%		

**Table 11:** Attenuation parameters obtained for amplitude and energy attenuation using the optimization method.

	Glass Rebar			Hybrid Rebar			Carbon Rebar		
	Mean	SD	CV	Mean	SD	CV	Mean	SD	CV
$\alpha_{A}$	0.0077	0.0011	13.7	0.0184	0.0062	33.8	0.0244	0.0084	34.3
$\alpha_{E}$	0.0035	0.0036	102.5	0.0187	0.014	72.5	0.0244	0.0049	20.1
$n_{A}$	0.178	0.054	30.1	0.106	0.033	30.9	0.144	0.173	120.3
$n_{E}$	0.311	0.098	31.6	0.132	0.159	120.5	0.142	0.176	124.0
$p_{A}$	1.37	0.24	17.6	1.58	0.18	11.7	1.79	0.02	0.9
$p_{E}$	1.55	0.21	13.7	1.33	0.40	29.9	1.68	0.23	13.7

## Data Availability

Data will be made available on request.
